# Whole transcriptome sequencing reveals the functional regulation of chicken reproduction by lncRNAs, miRNAs, and mRNAs across the hypothalamus-pituitary-ovary axis

**DOI:** 10.3389/fvets.2025.1687863

**Published:** 2025-10-22

**Authors:** Peifeng Li, Chengzhu Chu, Lijuan Hu, Genxi Zhang, Pengfei Wu, Qi Zhang

**Affiliations:** ^1^College of Animal Science, Shanxi Agricultural University, Taiyuan, China; ^2^College of Animal Science and Technology, Yangzhou University, Yangzhou, China; ^3^Tianjin Key Laboratory of Animal Molecular Breeding and Biotechnology, Tianjin Engineering Research Center of Animal Healthy Farming, Institute of Animal Science and Veterinary, Tianjin Academy of Agricultural Sciences, Tianjin, China

**Keywords:** non-coding RNAs, reproduction, Bian chicken, HPO axis, RNA-seq, animal breeding

## Abstract

The hypothalamic–pituitary-ovarian (HPO) axis serves as the pivotal regulatory system governing reproduction in chickens. This study performed whole transcriptome sequencing on hypothalamus, pituitary, and ovarian tissues of Bian chickens to identify differentially expressed (DE) lncRNAs, miRNAs, and mRNAs (*p* < 0.05, FC > 2) between low- and high-laying groups. The hypothalamus exhibited 57 DE lncRNAs, 86 DE miRNAs, and 36 DE mRNAs; the pituitary showed the highest numbers with 206 DE lncRNAs, 234 DE miRNAs, and 528 DE mRNAs; while the ovary contained 111 lncRNAs, 230 miRNAs, and 62 mRNAs. GO functional enrichment analysis indicated that trans-target genes of hypothalamic and pituitary DE lncRNAs were enriched in cell proliferation Biological process (BP) terms (e.g., cell cycle, mitotic cell cycle). Hypothalamic miRNA targets clustered in metabolic regulation (cellular metabolic process), whereas pituitary miRNAs governed transport processes (nitrogen compound transport, intracellular transport). DE mRNAs showed BP terms enrichment in serotonin biosynthesis process, pituitary gland development, and DNA integration. KEGG pathway enrichment analysis revealed that lncRNA targets were significantly enriched in Progesterone-mediated oocyte maturation and Oocyte meiosis pathways in both hypothalamus and pituitary, with additional enrichment in Cell cycle and DNA replication. Notably, miRNA target genes showed conserved enrichment in metabolic regulation-related pathways (Metabolic pathways, Cysteine and methionine metabolism) across all three tissues. Key enriched pathways for DE mRNAs included Steroid biosynthesis, Cortisol synthesis and secretion, and Hippo signaling pathway. Finally, we constructed lncRNA-mRNA and miRNA-mRNA pairwise interaction networks, as well as ceRNA regulatory networks, through which we identified key regulatory networks targeting critical DE mRNAs, including *GATA4, SMAD3, FOXL2, INHBA, POU1F1, LHX3, SPP1, SNAP25, COLQ,* and *AMPH*. These results elucidate the multi-tissue molecular mechanisms underlying egg-laying performance in chickens, providing novel targets for improving poultry reproductive efficiency through marker-assisted breeding.

## 1 Introduction

The hypothalamic–pituitary-ovarian (HPO) axis is a critical neuroendocrine network that regulates reproductive function in chickens (1, 2). The hypothalamus integrates environmental and internal signals, secreting gonadotropin-releasing hormone (GnRH), which then stimulates the anterior pituitary gland to synthesize and release luteinizing hormone (LH) and follicle-stimulating hormone (FSH) (3, 4). These gonadotropins subsequently act on the ovary, stimulating gametogenesis and the secretion of sex steroid hormones (5). Estradiol (E_2_), a key sex steroid produced by the ovaries, is essential for oocyte development, follicle maturation, and ovulation (6). Studies have shown that modern laying hens exhibit sustained pituitary sensitivity to GnRH, recurrent elevations in FSH mRNA levels, and cyclical rises in E_2_ levels, all of which support extended laying periods (7).

Beyond the core components of the HPO axis (GnRH, FSH, LH, steroid hormones), a complex interplay among other genes significantly influences reproductive function. One key player is *KiSS-1 metastasis-suppressor (KISS1)*, which encodes kisspeptin, a neuropeptide crucial for GnRH secretion and puberty onset (8). Mutations in *KISS1* or its receptor, *KISS1R* (also known as *GPR54*), can lead to hypogonadotropic hypogonadism. Furthermore, genes involved in neurokinin B (NKB) and dynorphin signaling, such as *tachykinin precursor 3 (TAC3)* and *prodynorphin (PDYN)*, respectively, interact with kisspeptin-expressing neurons in the arcuate nucleus, thus modulating GnRH pulsatility (9). Additionally, genes related to steroidogenesis, such as *cytochrome P450 family 19 subfamily A member 1 (CYP19A1)* and *hydroxy-delta-5-steroid dehydrogenase, 3 beta- and steroid delta-isomerase 2 (HSD3B2)*, which encode enzymes involved in estrogen and progesterone biosynthesis, are essential for follicular development and ovulation (10, 11). Li et al. (12) found that *7-dehydrocholesterol reductase (DHCR7)* plays a crucial role in chicken follicular development and selection downstream of estrogen signaling, influencing granulosa cell proliferation, apoptosis, and differentiation and thereby advancing follicle maturation. These studies highlight the intricate genetic network beyond the classical HPO axis hormones that contributes to reproductive health and fertility.

The HPO axis is also influenced by factors such as metabolic and nutritional status and environmental conditions at the organism level, and endocrine and metabolic regulation, cytokines, signaling pathways, and epigenetic modifications at the cellular and molecular levels (13). Furthermore, accumulating evidence suggests that non-coding RNAs are critically involved in this sophisticated regulatory network, potentially exerting significant influence on the HPO axis and its control of reproductive processes (14, 15). For instance, He et al. (16) found that *miR-7* selectively inhibits gonadotropin expression, synthesis, and secretion by targeting Raf1, and acts as a feedback switch regulated by GnRH (inhibitory) and estrogen (enhancing). In sheep, Leng et al. (1) found that lncRNA SM2, which is highly expressed in the pituitary, regulates cell proliferation and gonadotropin (FSH/LH) secretion by sponging *oar-miR-16b* and modulating TGF-β/SMAD2 signaling. Another lncRNA, MSTRG 4701.7, was also reported to competitively regulate proliferation and apoptosis in chicken follicular granulosa cells by targeting the miR-1786/RORα pathway (17).

While traditional methods such as microarray analysis and Northern blotting are widely used for identifying genes or ncRNAs, their application has significant limitations, including low throughput, the inability to detect novel transcripts, and their time-consuming nature. Moreover, they cannot provide comprehensive insights into complex ncRNA regulatory networks (18–20). RNA-seq offers high sensitivity and specificity, enabling the identification of novel transcripts and the quantification of gene expression levels across different conditions. Employing RNA-seq analysis of pituitary transcriptomes in sheep with high and low prolificacy, Zheng et al. (14) identified 57 lncRNAs and 298 mRNAs showing differential expression between the two conditions. These RNAs were found to be functionally enriched in pituitary hormone-related pathways and reproductive processes. Chen et al. (21) conducted a genome-wide analysis of pituitary-derived circRNAs in pigs at pre-, peri-, and post-puberty stages, and identified 5,148 circRNAs whose expression levels peaked during puberty onset and whose parental genes were enriched in pituitary-related pathways. Additionally, 17 differentially regulated circRNAs were found to be linked to miRNA-gene networks. These results provided insights into circRNA-mediated regulation of puberty timing in gilts. Fan et al. (22) analyzed expression profiles in Wuding chickens’ hypothalamus, pituitary, and ovary during laying and brooding, finding 590, 423, and 5,371 DE genes in these tissues, respectively. Li et al. (23) sequenced the pituitary transcriptome of Hy-Line Brown hens at 15, 20, 30, and 68 weeks of age, identifying 470 DE-lncRNAs, 38 DE-miRNAs, and 2,449 DE-mRNAs.

However, research on the ncRNA-mediated regulation of reproduction and egg production in the HPO axis of chickens remains limited. In this study, we sought to elucidate the involvement of HPO axis-linked ncRNAs in modulating reproductive performance in layer hens. Using Chinese indigenous Bian chickens as the experimental model, we collected hypothalamic, pituitary, and ovarian tissues from hens exhibiting extremely high and extremely low egg production phenotypes. Through whole-transcriptome sequencing and bioinformatic analysis, we aimed to systematically identify and characterize reproduction-related lncRNAs, miRNAs, and mRNAs within the HPO axis, thereby providing molecular insights into the regulatory mechanisms underlying avian reproduction.

## 2 Materials and methods

### 2.1 Animals

The Bian chicken is a prominent local breed in China, classified as a dual-purpose type for both meat and egg production. Originating from northern Shanxi Province and southern Inner Mongolia, the Bian chicken is characterized by its large egg weight, high-quality meat, and strong stress resistance. In 2011, the breed was included in the Animal Genetic Resources in China: Poultry. Bian chickens typically begin egg laying in September and reach peak production by early November. The total egg output per bird was recorded from September to November (228 days of age), a period encompassing the onset of laying, the increase to peak production, and one month of sustained peak lay. From this population, 15 healthy hens with the highest egg production and 15 with the lowest egg production were selected as the high-yield group (mean ± SD: 45.33 ± 2.75 eggs) and the low-yield group (mean ± SD: 26.80 ± 3.08 eggs) (*p* < 0.01, Supplementary Table S1). Within each group, hens were randomly allocated to three biological replicates, with five birds per replicate. Hypothalamic, pituitary, and ovarian tissues were collected from each bird, with pooled samples prepared for each replicate group. This resulted in six experimental sample sets: hypothalamus (HH), pituitary (HP), and ovary (HO) from high-yield birds, paired with the corresponding tissues (LH, LP, LO) from their low-yield counterparts. All chickens were obtained from the Bian Chicken Breeding Farm of Shanxi Agricultural University. The study protocol received ethical approval from the Institutional Animal Care and Use Committee of Shanxi Agricultural University (Approval No. SXAU-EAW-2023C. WW.011023199).

### 2.2 Sampling

All the chickens were euthanized via cervical dislocation. Subsequently, the head was removed, and an incision was made through the skull along the midline, exposing the brain. The hypothalamus was identified based on anatomical landmarks and then dissected using fine scissors and forceps. Immediately after the hypothalamus was collected, the pituitary gland was accessed by gently lifting it from the sella turcica at the base of the skull, and fine forceps were used to detach it from surrounding structures. For ovarian tissue collection, the abdominal cavity was opened to expose the left ovary. Cortical tissue was then isolated after carefully excising larger follicular structures, ensuring the purity of the collected samples.

### 2.3 RNA processing and sequencing

Total RNA was extracted from pooled tissue samples using TRIzol reagent (Invitrogen, Carlsbad, CA, United States) following the manufacturer’s protocol for animal tissues. RNA quality was evaluated using the Agilent 2100 Bioanalyzer (Agilent Technologies), yielding RNA Integrity Numbers (RINs) ≥ 8.0, and further confirmed by 1.5% denaturing agarose gel electrophoresis. RNA concentrations were quantified via NanoDrop spectrophotometry. For lncRNA and mRNA sequencing library construction, ribosomal RNA (rRNA) was depleted using the Ribo-off rRNA Depletion Kit (Vazyme, Nanjing, China). Subsequent strand-specific library preparation for lncRNA sequencing was performed with the VAHTS Universal V6 RNA-seq Library Prep Kit for Illumina (Vazyme), following the manufacturer’s guidelines.

Small RNA library construction was performed using the NEBNext Small RNA Library Prep Set for Illumina (NEB, MA, United States). Following total RNA extraction, small RNA fragments (18–30 nt) were isolated via polyacrylamide gel electrophoresis (PAGE) gel excision and recovery. Adaptors were sequentially ligated to the 3′ and 5′ ends, followed by reverse transcription and PCR amplification. The final library was purified by PAGE gel excision to recover ~140-bp fragments, which were dissolved in EB solution.

Quality control for library integrity and yield for both the lncRNA and small RNA libraries was conducted using the Agilent 2100 Bioanalyzer and the ABI StepOnePlus Real-Time PCR System (Life Technologies). Sequencing was performed on the Illumina HiSeq 4000 platform (Illumina, Inc., CA, United States) by Gene Denovo Biotechnology Co. (Guangzhou, Guangdong, China).

### 2.4 Sequencing data collection and analysis

Raw reads were filtered using fastp (24) to remove adapter sequences and low-quality bases, generating high-quality clean reads for downstream transcriptomic analyses. Bowtie2 (25) was used to map the clean reads obtained from the lncRNA sequencing library to the ribosomal RNA (rRNA) database, following which rRNA-aligned reads were removed. The remaining unmapped clean reads were subsequently aligned to the reference genome (GRCg6a) using HISAT2 (v2.1.0) (26) and assembled into transcripts with StringTie (v1.3.1) (27). These procedures enabled the identification of both annotated and novel transcripts. Novel transcripts were evaluated for coding potential using CNCI (28) and CPC (29), and those predicted to be non-coding by both tools were retained as novel lncRNAs. Finally, the *trans*-target genes of lncRNAs were predicted using a rigorous screening criterion established based on correlation coefficients ≥0.98 between lncRNA and mRNA co-expression.

Blastall (v2.2.25) was first used to align small RNA clean tags to the GenBank (Release 209.0) and Rfam (Release 11.0) databases to exclude rRNA/scRNA/snoRNA/snRNA/tRNA contamination. Then, the clean reads were mapped to the reference genome using Bowtie (30) to remove exonic/intronic (potential mRNA degradation fragments) and repetitive sequences. The remaining clean tags were annotated against miRBase (release 22) to identify conserved miRNAs. Mirdeep2 software (31) was used to predict novel miRNAs by exploring the secondary structure, the Dicer cleavage site, and the minimum free energy of the unannotated small RNA tags. Candidate mRNA target genes were predicted with both Miranda (v3.3a) and TargetScan (v7.0), with only the intersecting hits commonly predicted by both tools selected for subsequent analysis. Differential expression analysis for mRNAs and lncRNAs was separately conducted using DESeq2 (32), while differential miRNA expression was analyzed with edgeR (33). Finally, Gene Ontology (GO) function and KEGG pathway enrichment analyses of target genes corresponding to the differentially expressed (DE) lncRNAs, miRNAs, and mRNAs were performed using the clusterProfiler R package.

## 3 Results

### 3.1 Quality control of sequencing data

A total of 1,639,957,796 raw reads were generated through lncRNA sequencing (Supplementary Table S2). After quality filtering, 1,635,297,404 clean reads were retained, corresponding to an overall clean read rate of 99.71%. Each sample achieved a clean data ratio exceeding 99.63%. Sequencing quality metrics showed Q30 values above 91.51%, and GC contents ranging between 44.00 and 46.15%, consistent with RNA sequencing expectations. These results indicated that the lncRNA sequencing data were of good quality and suitable for downstream analyses.

For miRNA sequencing, a total of 645,744,492 raw reads were generated, yielding 642,197,641 high-quality reads (99.45% of raw reads) after the removal of low-quality sequences (Supplementary Table S3). Following stringent filtering to remove reads lacking 3′ adapters, empty reads without detectable small RNA inserts, reads contaminated with 5′ adapters, and polyA-tailed reads (>70% adenine content), 629,596,293 clean reads were retained, corresponding to 98.03% of the high-quality reads. Sample-specific clean tags retention rates ranged from 94.58 to 98.60%, demonstrating that data processing was efficient and reliable.

### 3.2 Sequencing alignment results

Analysis of lncRNA sequencing alignment results demonstrated that rRNA contamination control was effective, with mapped rRNA reads accounting for less than 1.07% across all samples, while unmapped rRNA reads (valid reads) exceeded 99.16% (Supplementary Table S4). Among the valid reads, 94.37–95.67% were successfully mapped to the reference genome, indicative of high alignment efficiency. Regarding annotation regions, reads were predominantly enriched in exons (48.49–69.93%), with partial distribution in introns (25.46–43.99%) and intergenic regions (4.48–8.92%), consistent with the transcriptional characteristics of lncRNAs.

For miRNA, sequencing alignment results showed an average total clean tag count (Total_Abundance) of 629.60 million (Supplementary Table S5), with tags in miRBase of chicken accounting for 51.99–74.93% (Exist_miRNA_Abundance). Tags aligned to other vertebrates’ miRBase constitute 4.51–10.20% (Known_miRNA_Abundance) of the total clean tag count, and novel miRNAs represent 0.05–0.09% (Novel_miRNA_Abundance). Base-edited miRNAs (Exist_miRNA_Edit_Abundance) comprised 13.59–23.65% (average 18.22%) of the total miRNAs, indicating that editing events were prevalent.

### 3.3 Differential expression analysis

**Figure 1:**
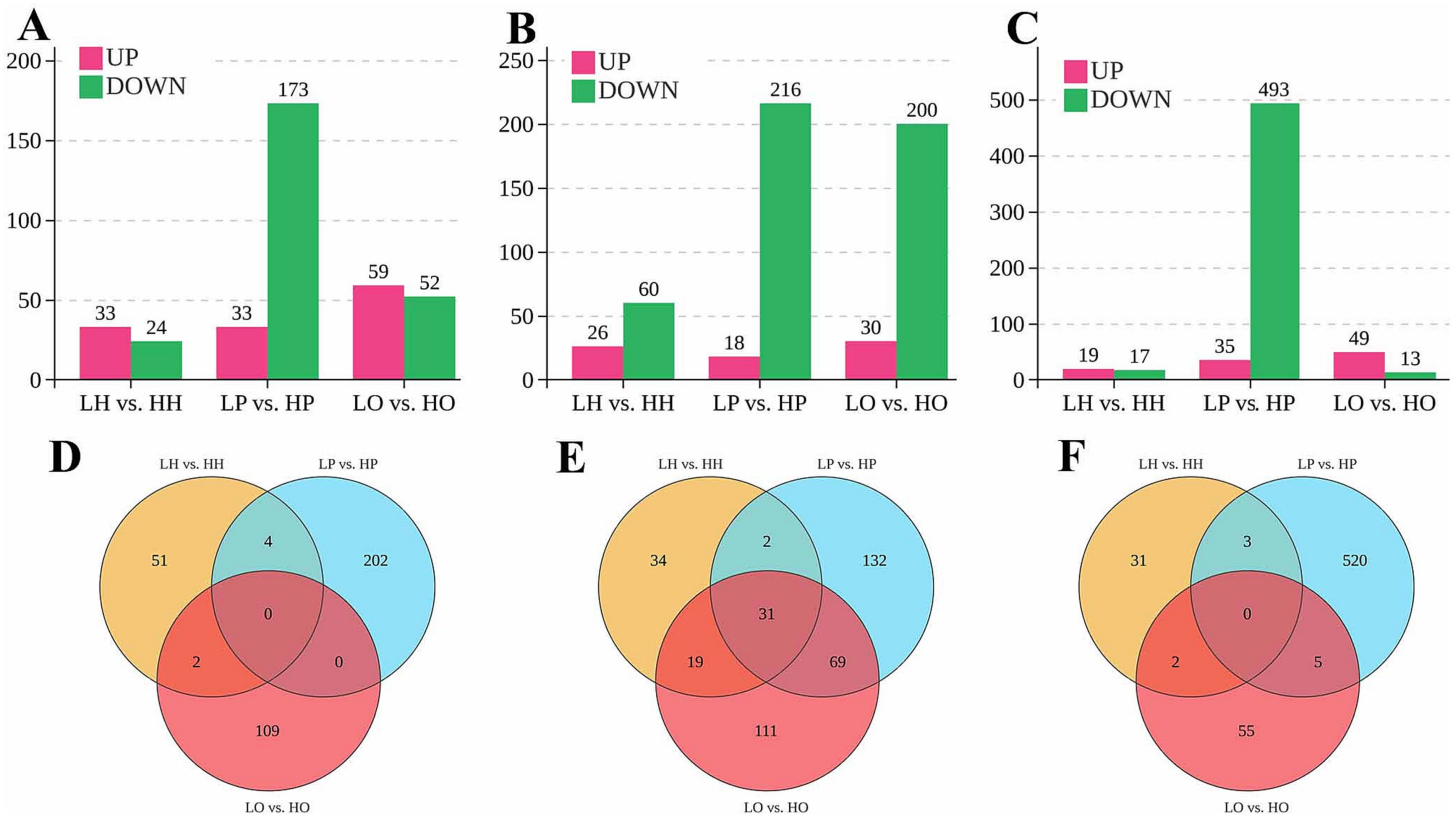
Results of lncRNA, miRNA, and mRNA differential expression analysis. **(A)** The number of differentially expressed (DE) lncRNAs in hypothalamus, pituitary and ovary; **(B)** The number of DE miRNAs in hypothalamus, pituitary and ovary; **(C)** The number of DE mRNAs in hypothalamus, pituitary and ovary; **(D)** Venn diagram of DE lncRNAs; **(E)** Venn diagram of DE miRNAs; **(F)** Venn diagram of DE mRNAs.

DE lncRNAs, miRNAs, and mRNAs between the low-yield and high-yield groups in hypothalamic, pituitary, and ovarian tissues of Bian chicken were identified using the criteria of *p* < 0.05 and fold change (FC) > 2 (Figures 1A–C). Compared to the low-yield group, the high-yield group exhibited 57 DE lncRNAs in the hypothalamus (33 upregulated, 24 downregulated), along with 86 DE miRNAs (26 upregulated, 60 downregulated), and 36 DE mRNAs (19 upregulated, 17 downregulated). The pituitary displayed the most pronounced changes, with 206 DE lncRNAs (33 upregulated, 173 downregulated), 234 DE miRNAs (18 upregulated, 216 downregulated), and 528 DE mRNAs (35 upregulated, 493 downregulated), which showed a strong bias toward downregulation. In the ovary, we identified 111 DE lncRNAs (59 upregulated, 52 downregulated), 230 DE miRNAs (30 upregulated, 200 downregulated), and 62 DE mRNAs (49 upregulated, 13 downregulated). Notably, this tissue featured the highest number of upregulated lncRNAs and mRNAs.

Venn diagrams were used to visualize the distribution of DE RNAs between low- and high-yield groups across the hypothalamus (LH *vs*. HH), pituitary (LP *vs*. HP), and ovary (LO *vs*. HO). For lncRNAs, the pituitary featured the highest unique transcript count (202 lncRNAs), followed by the ovary with 109 and the hypothalamus with 51 (Figure 1D). Four lncRNAs were shared between the hypothalamus and the pituitary, and two were shared between the hypothalamus and the ovary. None was shared between the pituitary and the ovary or across all three tissues. Regarding miRNAs, the pituitary contained the highest number of unique miRNAs (132 miRNAs), followed by the ovary (111 miRNAs) and the hypothalamus (34 miRNAs) (Figure 1E). Thirty-one miRNAs were common to all tissues. Additionally, two miRNAs were common to the hypothalamus and the pituitary, 19 were common to the hypothalamus and the ovary, and 69 were shared between the pituitary and the ovary. For mRNAs, the pituitary exhibited the greatest unique transcript count (520 mRNAs), while the hypothalamus had 31 and the ovary 55 (Figure 1F). The pairwise overlaps included 3 mRNAs shared between the hypothalamus and the pituitary, 2 shared between the hypothalamus and the ovary (2 mRNAs), and 5 shared between the pituitary and the ovary; no mRNAs were common to all tissues.

### 3.4 GO functional enrichment analysis

**Figure 2:**
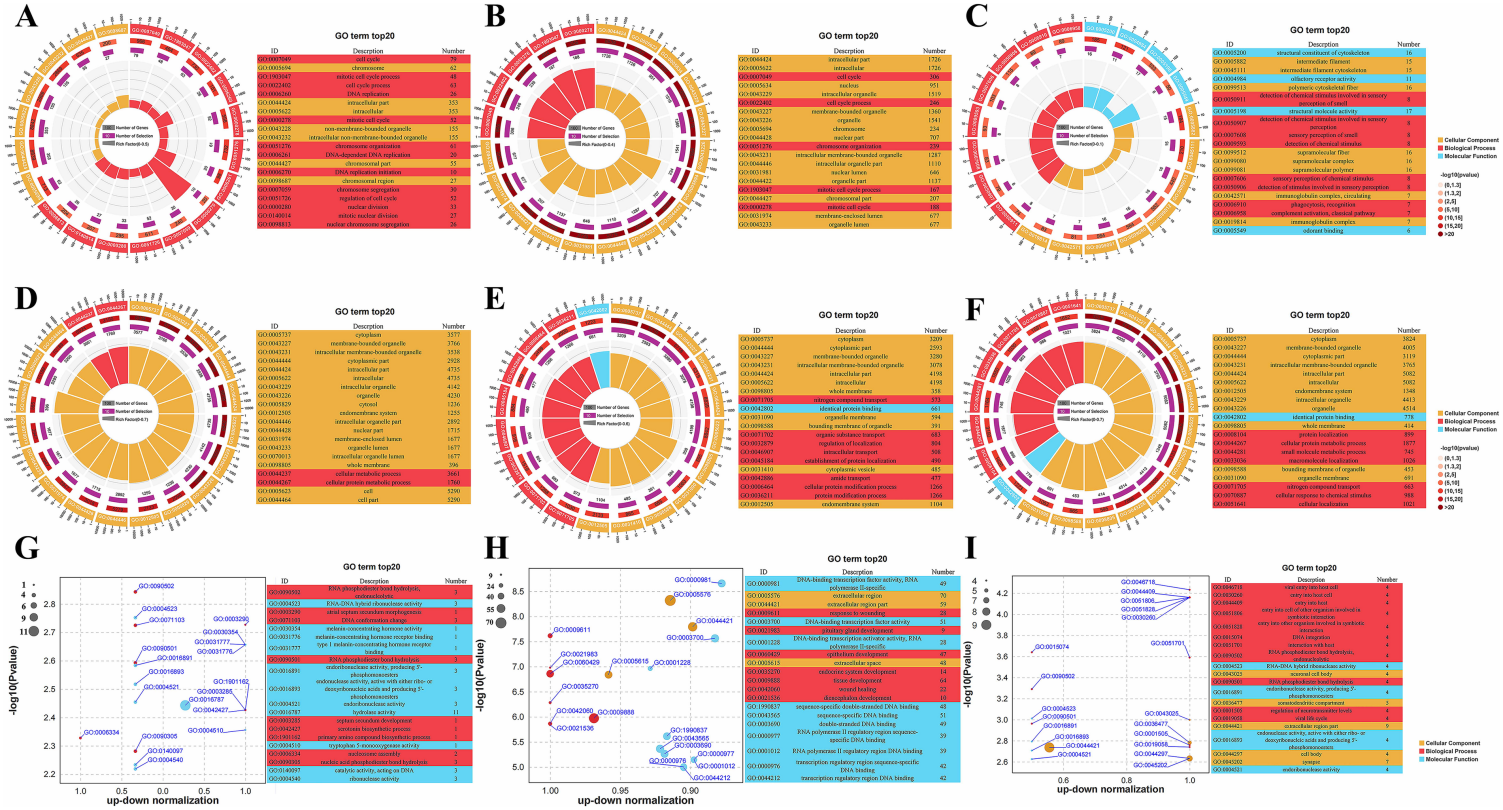
GO functional enrichment analysis results. **(A–C)** Circular plot of top 20 GO results for trans-target genes of DE lncRNAs in the hypothalamus, pituitary and ovary; **(D–F)** Circular plot of top 20 GO results for target genes of DE miRNAs in the hypothalamus, pituitary and ovary; **(G–I)** Bubble plot of top 20 GO results for DE mRNAs in the hypothalamus, pituitary and ovary.

A total of 545 lncRNA *trans*-target genes were identified in the hypothalamus, 2,783 in the pituitary gland, and 97 in the ovaries. Meanwhile, across the whole genome, a total of 8,199, 7,438, and 8,936 miRNA target genes were predicted in the hypothalamus, pituitary gland, and ovaries, respectively. To further elucidate their functional roles, GO enrichment analysis was performed on the *trans*-target genes of the DE lncRNAs, the target genes of the DE miRNAs, and the DE mRNAs. The top 20 GO terms are shown in Figure 2 as both circle and bubble charts.

GO enrichment analysis of the DE lncRNA target genes across the hypothalamus, pituitary, and ovary revealed distinct tissue-specific patterns (Figures 2A–C; Supplementary Table S6). In both the hypothalamus and the pituitary gland, lncRNA target genes were significantly enriched in cell cycle-related terms within the GO Biological Process (BP) category, such as cell cycle, cell cycle process, mitotic cell cycle process, and mitotic cell cycle, indicating that lncRNAs in these tissues play a crucial role in the regulation of cell proliferation and division. In contrast, lncRNA target genes in the ovary were enriched in processes related to sensory perception, such as detection of chemical stimulus involved in sensory perception and detection of chemical stimulus, implying that they function in sensory signal transduction.

GO enrichment analysis of the DE miRNA target genes revealed tissue-specific functions (Supplementary Table S7). In the hypothalamus (Figure 2D), the miRNA targets were primarily associated with metabolic processes (cellular metabolic process and cellular protein metabolic process), suggesting that they play regulatory roles in cellular metabolism. The miRNA target genes in the pituitary gland were enriched in transport and localization processes, including nitrogen compound transport, intracellular transport, and regulation of localization, indicating that they are involved in nutrient and protein trafficking (Figure 2E). In the ovary (Figure 2F), meanwhile, the miRNA target genes showed enrichment in protein localization, metabolic processes, and cellular responses to chemical stimuli, with terms such as protein localization, small molecule metabolic process, and cellular response to chemical stimulus being highly represented.

GO enrichment analysis of the DE mRNAs across the hypothalamus, pituitary, and ovary revealed that they participated in a diverse range of biological processes (Supplementary Table S8). In the hypothalamus (Figure 2G), the DE mRNAs were found to be involved in RNA processing and neurotransmitter synthesis, as evidenced by their enrichment in RNA phosphodiester bond hydrolysis, endonucleolytic, and serotonin biosynthetic process. The DE mRNAs in the pituitary gland were enriched in developmental processes, including pituitary gland development, endocrine system development, and tissue development (Figure 2H). In the ovary (Figure 2I), the DE mRNAs were predominantly linked to processes such as DNA integration, RNA phosphodiester bond hydrolysis, endonucleolytic, and regulation of neurotransmitter levels. These results suggested that ovarian mRNAs are involved in complex interactions with pathogens and may also contribute to genetic information processing and neurological function.

In summary, a systematic analysis of GO enrichment results across the hypothalamus, pituitary, and ovary revealed that lncRNAs, miRNAs, and mRNAs exhibit tissue-specific enrichment profiles, reflecting their diverse biological functions and regulatory mechanisms. These findings provide valuable insights into the molecular underpinnings of tissue-specific physiological processes.

### 3.5 KEGG pathway enrichment analysis

**Figure 3:**
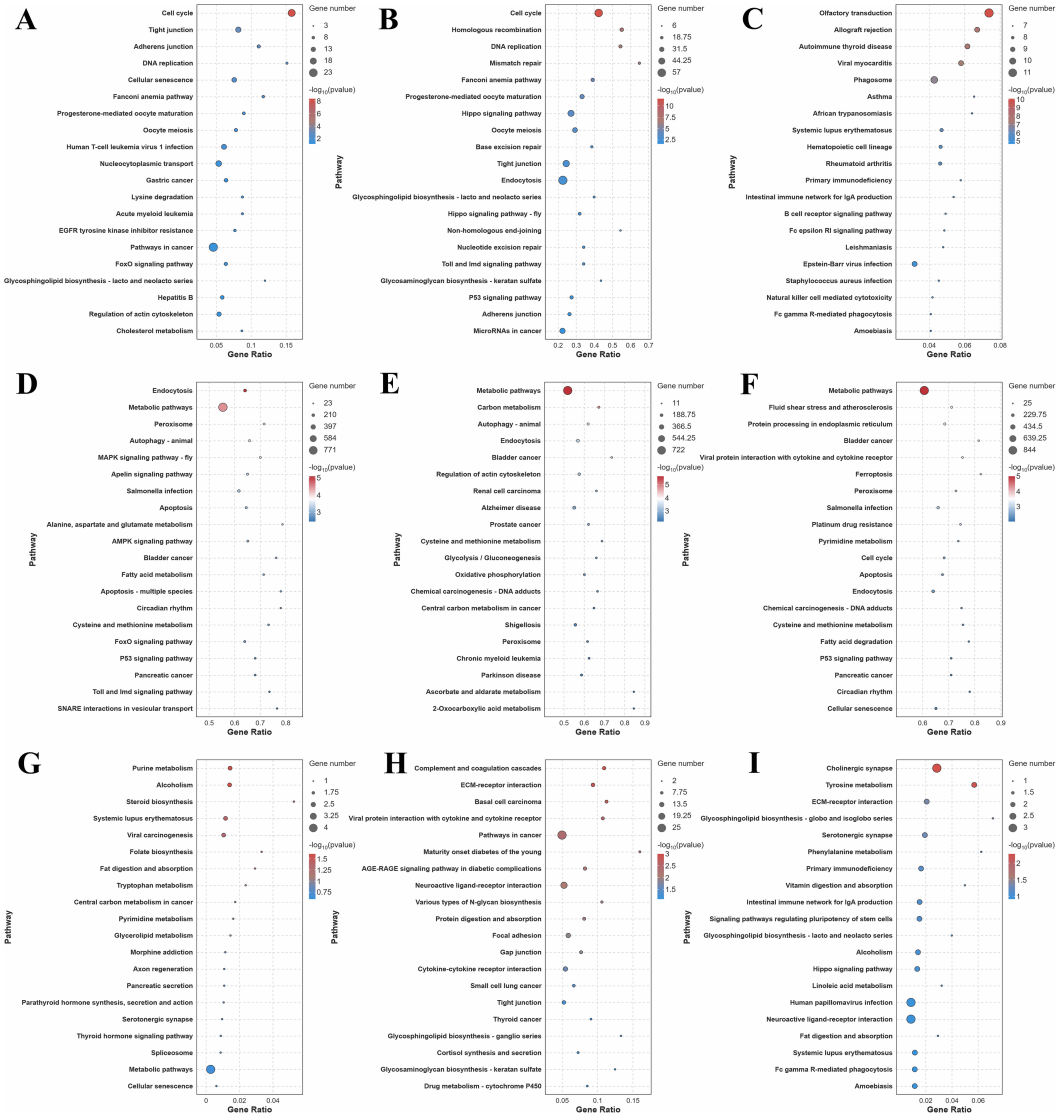
KEGG pathway enrichment analysis results. **(A–C)** Top 20 KEGG results for trans-target genes of DE lncRNAs in the hypothalamus, pituitary and ovary; **(D–F)** Top 20 KEGG results for target genes of DE miRNAs in the hypothalamus, pituitary, and ovary; **(G–I)** Top 20 KEGG results for DE mRNAs in the hypothalamus, pituitary, and ovary.

In the hypothalamus and pituitary, DE lncRNA target genes were significantly enriched in direct reproduction-associated pathways (Figures 3A,B; Supplementary Table S9). In both the hypothalamus and the pituitary, these genes exhibited strong enrichment in Progesterone-mediated oocyte maturation and oocyte meiosis, processes that are critical for follicular development and ovulation. LncRNA target genes in the hypothalamus and pituitary were also prominently associated with other pathways, such as Cell cycle, DNA replication, and Adherens junction, suggesting that they play roles in cellular proliferation and signal transduction during reproduction. In contrast, ovarian DE lncRNA target genes were predominantly linked to immune/inflammatory pathways, reflecting their involvement in tissue-specific immune regulation of follicle integrity (Figure 3C).

In the hypothalamus, pituitary, and ovary, DE miRNA target pathways were found to be involved in both shared and tissue-specific regulation of reproductive processes (Figures 3D–F; Supplementary Table S10). Four pathways showed enrichment across all three tissues, namely, Metabolic pathways, Peroxisome, Endocytosis, and Cysteine and methionine metabolism. The universal enrichment of these pathways reflected fundamental requirements for energy metabolism, redox balance, signal transduction, and amino acid homeostasis across reproductive tissues. The hypothalamus and the pituitary exhibited unique co-enrichment in autophagy-animal, reflecting shared mechanisms for the maintenance of proteostasis in neuroendocrine cells under metabolic stress. The hypothalamus and the ovary exhibited exclusive convergence on reproductive-regulatory pathways, including Apoptosis, Circadian rhythm, and p53 signaling. The miRNA target pathways in the pituitary and the ovary showed unique co-enrichment in Chemical carcinogenesis-DNA adducts, emphasizing their mutual vulnerability to DNA damage and shared repair mechanisms in rapidly dividing secretory and follicular cells. For tissue-specific pathways, the hypothalamus featured neuroendocrine-specific regulators, including MAPK signaling pathway-fly, Apelin signaling, and FoxO signaling. The pituitary was distinguished by carbohydrate metabolism (Carbon metabolism, Glycolysis/Gluconeogenesis) and cytoskeletal organization (Regulation of actin cytoskeleton), aligning with its high energy demands for hormone secretion and exocytotic machinery. The ovary uniquely engaged Ferroptosis, Cell cycle, and Protein processing in endoplasmic reticulum, complemented by cellular senescence pathways relevant to reproductive aging.

A comparative analysis of differential mRNA-enriched KEGG pathways in the hypothalamus, pituitary, and ovary revealed both distinct and overlapping associations with reproduction (Figures 3G–I; Supplementary Table S11). DE mRNAs in the hypothalamus and the ovary were enriched in several shared pathways, including the direct reproduction-related Serotonergic synapse, which modulates neuroendocrine signals in the hypothalamus and influences ovarian function. Additional shared enrichment included pathways such as Alcoholism, Systemic lupus erythematosus, and Fat digestion and absorption, which can indirectly affect reproduction through metabolic and immune dysfunction. The DE mRNAs in the pituitary and the ovary shared enrichment in two pathways, namely, ECM-receptor interaction (indirectly related via cell-matrix interactions) and the directly relevant Neuroactive ligand-receptor interaction, which is critical for hormone signaling in both tissues. In the hypothalamus, meanwhile, specific enrichment was observed for steroid biosynthesis and Parathyroid hormone synthesis, secretion, and action. Pituitary-specific DE mRNAs were enriched in Cortisol synthesis and secretion, a pathway that directly modulates the hypothalamic–pituitary-gonadal (HPG) axis by fine-tuning reproductive hormone release. In the ovary, DE mRNAs showed exclusive enrichment in signaling pathways regulating pluripotency of stem cells and the Hippo signaling pathway, highlighting their importance in gametogenesis.

### 3.6 Interaction network analysis of the differentially expressed RNAs

**Figure 4:**
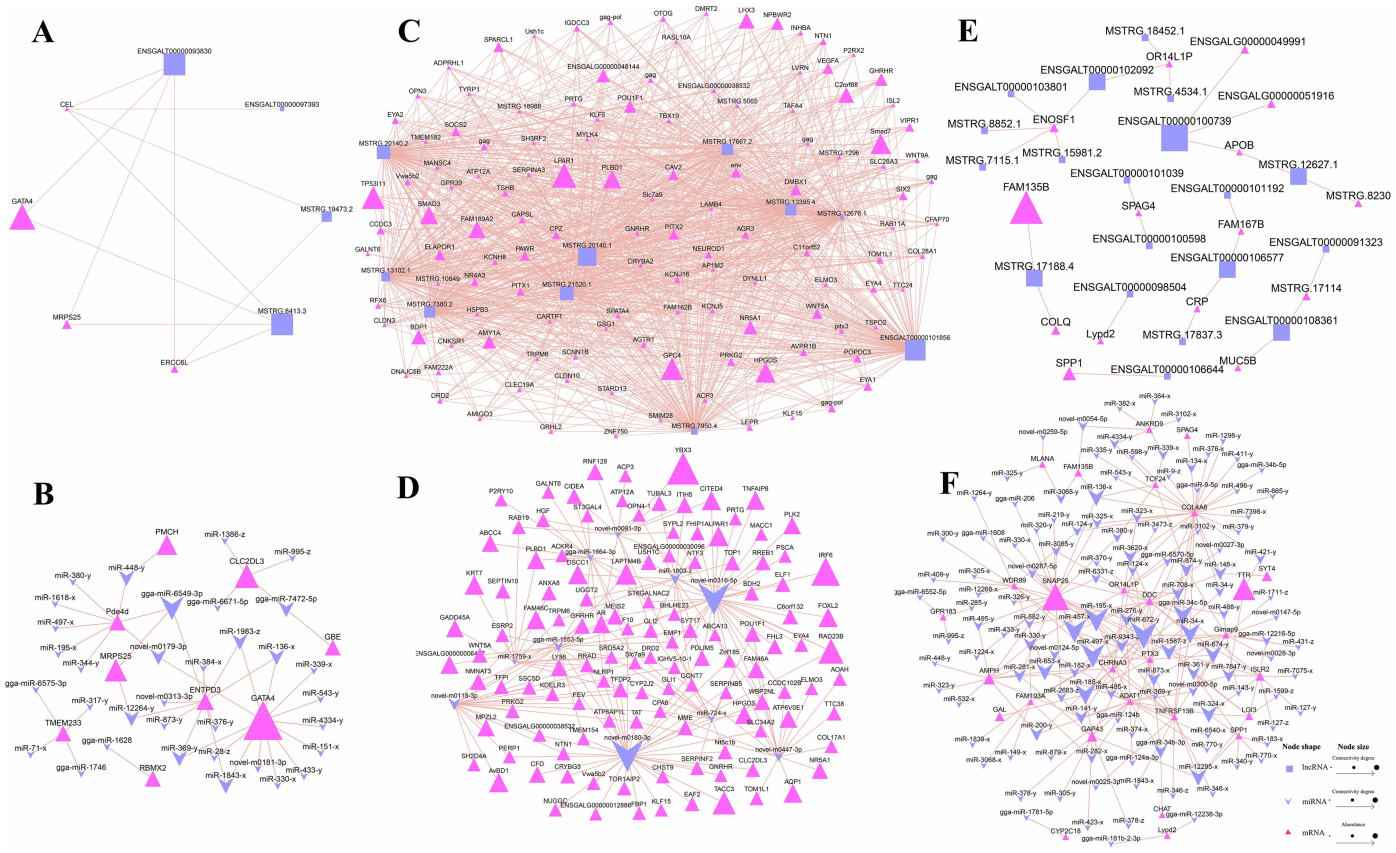
Differentially expressed (DE) RNA interaction network diagram. **(A,C,E)** Network of DE lncRNA-DE mRNA in the hypothalamus, pituitary, and ovary; **(B,D,F)** Network of DE miRNA-DE mRNA in the hypothalamus, pituitary, and ovary.

To further understand the interactions among the DE RNAs, we constructed interaction networks between DE lncRNAs and *trans*-DE mRNAs, as well as between DE miRNAs and DE mRNAs. In these networks, lncRNA and miRNA node sizes represented their connectivity degree, while mRNA node sizes represented their expression abundance. In the hypothalamic network (Figures 4A,B), the *GATA4* gene exhibited the highest abundance. Furthermore, it connected to the lncRNAs *ENSGALT00000093830* and *MSTRG.8413.3*, both sharing the highest degree of connectivity (Figure 4A). *GATA4* also interacted with 13 miRNAs, including *miR-136-x*, *miR-1983-z*, and *miR-1843-x* (Figure 4B).

A substantial number of DE lncRNA-DE mRNA and DE miRNA-DE mRNA pairs were generated in the pituitary, and the top 10 lncRNAs and miRNAs with the highest connectivity were selected for visualization. Figure 4C shows that among the highly abundant genes, *SMAD family member 3 (SMAD3), family with sequence similarity 189 member A2 (FAM189A2), LIM homeobox 3 (LHX3)* and *phospholipase B domain containing 1 (PLBD1)* were potentially associated with pituitary-regulated reproduction. Further analysis revealed that they all interacted with the top 10 most abundant DE lncRNAs. Within the miRNA-mRNA interaction network (Figure 4D), *Y-box binding protein 3 (YBX3)* showed the highest expression abundance as a target of novel-m0091-3p. Additionally, the highly expressed genes *lysosomal protein transmembrane 4 beta (LAPTM4B)* and *forkhead box L2 (FOXL2)*, potentially implicated in pituitary function regulation, were identified as targets of *gga-miR-1664-3p* and *novel-m0316-5p*, respectively. Notably, *novel-m0316-5p* ranked among the miRNAs with the highest connectivity in the network.

Finally, in the DE lncRNA-DE mRNA interaction network in ovarian tissue, the most abundant mRNA was *similarity 135 member B (FAM135B)*, followed by *secreted phosphoprotein 1 (SPP1)* and *collagen like tail subunit of asymmetric acetylcholinesterase (COLQ)* (Figure 4E). All three genes are potentially involved in ovarian development and reproduction. In the DE miRNA-DE mRNA network in the same tissue, we identified *synaptosome associated protein 25 (SNAP25)* as the most abundant gene, and it was targeted by *miR-497-x*, *miR-457-x*, and *miR-672-y*, with the latter exhibiting the highest connectivity (Figure 4F). Additionally, the *AMPH* gene, potentially involved in the regulation of ovarian reproduction, was identified as a target of several miRNAs, including *miR-653-x* and *miR-200-y*.

### 3.7 Competing endogenous RNA regulatory network analysis

**Figure 5:**
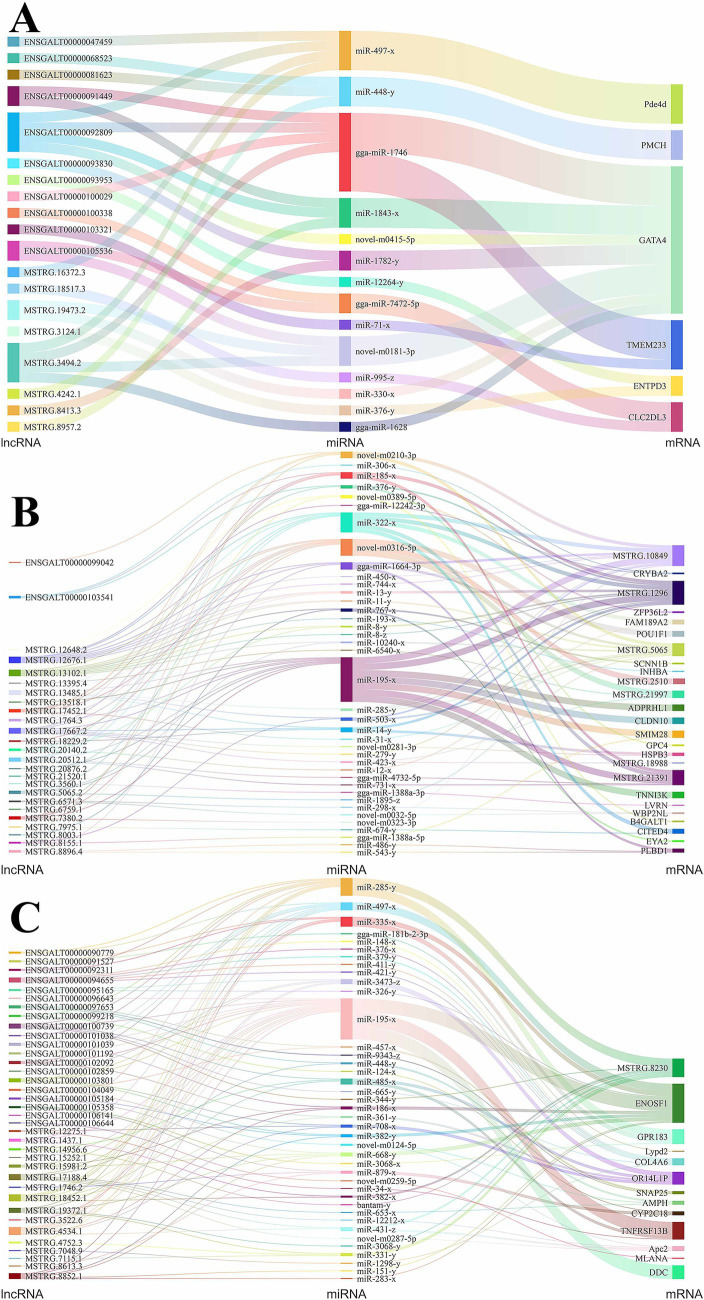
CeRNA interaction network diagram of DE lncRNAs, DE miRNAs, and DE mRNAs. **(A)** Sankey diagram of ceRNA relationship for hypothalamus; **(B)** Sankey diagram of ceRNA relationship for Pituitary; **(C)** Sankey diagram of ceRNA relationship for ovary.

The competing endogenous RNA (ceRNA) regulatory mechanism represents a key interaction framework among lncRNAs, miRNAs, and mRNAs, serving as an important pathway through which miRNAs and lncRNAs exert their functional roles. Consequently, we undertook a comprehensive ceRNA regulatory network analysis involving DE lncRNAs, DE miRNAs, and DE mRNAs in hypothalamic, pituitary, and ovarian tissues, and constructed corresponding Sankey diagrams to visualize these regulatory relationships. In those figures, lncRNA, miRNA and mRNA sizes represented their connectivity degree. In the hypothalamus, we identified 30 ceRNA regulatory relationship pairs (Figure 5A), involving 19 DE lncRNAs, 14 DE miRNAs, and 6 DE mRNAs. *GATA4* exhibited the highest degree of connectivity among the mRNAs within the regulatory network, suggesting that it may play a potentially critical role in hypothalamic function. It was modulated upstream by *gga-miR-1746*, which showed the greatest connectivity among the miRNAs. This miRNA interacts with multiple lncRNAs, including *ENSGALT00000092809* and *MSTRG.3494.2*. Additionally, we found that lncRNAs targeting the *miR-448-y-PMCH* axis may also influence ovarian development and function by modulating hypothalamic activity.

In the pituitary tissue, a total of 14,935 ceRNA regulatory relationship pairs were predicted based on the targeting relationships among DE lncRNAs, DE miRNAs, and DE mRNAs. Subsequently, we selected the top 100 pairs based on lncRNA-mRNA expression correlation for visualization, as shown in Figure 5B. The genes *POU class 1 homeobox 1 (POU1F1)* and *inhibin beta A subunit (INHBA)* are closely associated with pituitary regulatory function, and they were predicted to be targeted by the miRNAs *novel-m0316-5p* and *miR-193-x*, respectively. *Novel-m0316-5p* exhibited targeting relationships with the lncRNAs exhibiting the highest connectivity, namely, *MSTRG.12676.1* and *MSTRG.13102.1*, while *miR-193-x* was only predicted to target lncRNA *MSTRG.13102.1*. Additionally, three genes *MSTRG.1296*, *MSTRG.10849*, and *MSTRG.21391*, which displayed the greatest degree of connectivity, may also influence pituitary function, suggesting that ceRNA-targeted lncRNA-miRNA pairs that regulate the expression of these three genes may play significant roles in this process.

In ovarian tissue, we similarly constructed a ceRNA regulatory network comprising the top 100 DE lncRNA-DE mRNA pairs showing the strongest expression correlation (Figure 5C). This network included 36 DE lncRNAs, 40 DE miRNAs, and 13 DE mRNAs. The gene *synaptosome associated protein 25 (SNAP25)* directly influences ovarian function, making the *ENSGALT00000104049/ENSGALT00000097653-miR-457-x-SNAP25* and *ENSGALT00000097653-miR-9343-z-SNAP25* ceRNA regulatory axes particularly significant. M*iR-195-x* exhibited the highest degree of connectivity among the miRNAs, targeting the *mitochondrial enolase superfamily member 1 (ENOSF1), TNF receptor superfamily member 13B (TNFRSF13B),* and *dopa decarboxylase (DDC)* genes, all of which may participate in ovarian function regulation. Upstream, *miR-195-x* showed targeting relationships with 14 highly connected lncRNAs, including *MSTRG.4534.1*, *MSTRG.18452.1*, and *ENSGALT00000100739*.

## 4 Discussion

Eggs are a globally significant food source, providing high-quality protein, essential vitamins (e.g., A, D, E, and B), and minerals (e.g., iron and selenium) crucial for human health and development (34–36). Their nutritional value makes them particularly important in combating malnutrition, especially in developing countries (37, 38). However, egg production in poultry is a complex process influenced by numerous factors, including genetics, nutrition (feed quality and availability), environmental conditions (temperature, light, and housing), the bird’s overall health, and management practices (34, 39–41). These factors collectively impact the physiological mechanisms that regulate reproduction in hens. At the core of these mechanisms is the intricate interplay between hormones and neural signals, which govern egg development and release (42). Central to this process is the HPO axis, a neuroendocrine system that orchestrates reproductive function (13).

To further unravel the molecular mechanisms by which the HPO axis regulates egg production, we employed whole-transcriptome sequencing of hypothalamic, pituitary, and ovarian tissues collected from high-yield and low-yield Bian chickens, systematically profiling the expression patterns of lncRNAs, miRNAs, and mRNAs. In this study, tissues from five hens were pooled, forming one biological replicate. While this approach may reduce statistical power and obscure individual variations, rigorous quality control and standardized protocols ensured that the pooled samples reliably reflected population-level characteristics. Differential expression analysis across hypothalamic, pituitary, and ovarian tissues revealed distinct regulatory patterns for lncRNAs, miRNAs, and mRNAs between low- and high-yield groups. The pituitary exhibited the highest total number of DE RNAs (206 lncRNAs, 234 miRNAs, and 528 mRNAs), with downregulated molecules predominating in the high-yield group. In contrast, the ovary showed the most upregulated RNA species (59 lncRNAs, 30 miRNAs, and 49 mRNAs). The hypothalamus had the lowest number of DE molecules (57 lncRNAs, 86 miRNAs, and 36 mRNAs).

Analysis of the top 20 GO terms showed that lncRNA functions exhibit profound tissue-specific specialization. In neuroendocrine tissues (hypothalamus, pituitary), lncRNAs uniformly regulated cell proliferation, as indicated by their observed enrichment in the BP terms cell cycle, cell cycle process, mitotic cell cycle process, and mitotic cell cycle. Cell proliferation and mitosis are indispensable for the development, functional integration, and homeostasis of the hypothalamus and pituitary gland, which support both the precise differentiation of endocrine cell types and the dynamic adaptation to physiological demands required for proper regulation of critical neuroendocrine functions (43, 44). In the hypothalamus, miRNAs showed the broadest metabolic control, specifically in cellular metabolic process and cellular protein metabolic process. This is consistent with the role of the hypothalamus as a metabolic integrator crucial for physiological functions such as reproduction, energy balance, and thermoregulation (45, 46). DE Pituitary miRNAs were uniquely enriched in transport-related processes, including nitrogen compound transport and intracellular transport. These processes are closely linked to pituitary endocrine secretion via the regulation of hormone synthesis, vesicle trafficking, and calcium-dependent exocytosis, which are essential for the proper release of hormones such as LH and GH (47–49). In the hypothalamus, DE mRNAs were predominantly enriched in neurodevelopmental processes (serotonin biosynthesis process) and nucleic acid metabolism (RNA phosphodiester bond hydrolysis, endonucleolytic), aligning with its neuromodulatory functions (50, 51). In the pituitary, meanwhile, the DE mRNAs were mainly associated with developmental morphogenesis (pituitary gland development, endocrine system development, and tissue development). In the ovary, enrichment analysis of DE lncRNA target genes, DE miRNA target genes, and DE mRNAs also highlighted several important BP terms, such as detection of chemical stimulus, protein localization, nitrogen compound transport, and RNA phosphodiester bond hydrolysis, endonucleolytic. The interplay among these processes reflects the ovary’s capacity for dynamic adaptation to environmental signals and precise metabolic regulation (52).

KEGG pathway enrichment analysis revealed that the *trans*-target genes of the DE lncRNAs were enriched in the Progesterone-mediated oocyte maturation and oocyte meiosis pathways in both the hypothalamus and the pituitary. Progesterone-mediated oocyte maturation is a key step in the reproductive cycle, ensuring that the oocyte reaches a state of readiness for fertilization. Progesterone plays a dual role in this process through both genomic and non-genomic signaling pathways (53, 54). Oocyte meiosis is a highly regulated process essential for the production of haploid gametes, which safeguards genetic diversity and enables successful fertilization (54, 55). Additional pathways enriched in both the hypothalamus and the pituitary included Cell cycle, DNA replication, and Adherens junctions. Cell cycle and DNA replication pathways are related to cell proliferation, analogous to the previously mentioned enriched GO BP terms. Adherens junctions are cell structures crucial for cell–cell adhesion, tissue organization, and signal transduction (56). They likely contribute to the proper functioning of the hypothalamus and the pituitary by maintaining tissue integrity, influencing signaling pathways, and regulating cell polarity (57), all of which are essential for hormone production and the regulation of the HPO axis.

In the hypothalamus, the pituitary, and the ovary, KEGG enrichment analysis relating to DE miRNA target genes revealed several key overlapping pathways, such as Metabolic pathways, Endocytosis, Peroxisome, and Cysteine and methionine metabolism. Metabolic pathways are fundamental for cellular energy and biosynthetic processes, supporting the high energy demands of reproductive processes (58, 59). Endocytosis is important for receptor-mediated signaling, which is involved in hormone regulation (60, 61). Peroxisome pathways contribute to both lipid and reactive oxygen species metabolism, processes important for steroidogenesis and follicular development (62, 63). Cysteine and methionine metabolism, a component of amino acid metabolism, influences antioxidant capacity and methylation processes, thereby affecting reproductive functions (64–66). Shared enrichment between the hypothalamus and the ovary was also observed for Apoptosis, p53 signaling pathway, and Circadian rhythm. Apoptosis and p53 signaling are key regulators of follicular atresia and germ cell quality control (67–69), while the circadian rhythm pathway influences the timing of reproductive events (70, 71). We also identified unique enrichment patterns in individual tissues, such as the FoxO signaling pathway in the hypothalamus, Carbon metabolism in the pituitary, and Ferroptosis in the ovary.

In the KEGG enrichment results for DE mRNAs, pathways co-enriched across all three tissues were limited, while pairwise overlaps uncovered integrated communication axes. Notably, DE mRNAs in the hypothalamus and ovary were both enriched in pathways with dual relevance, most notably Serotonergic synapse, which directly regulates neuroendocrine signaling in the hypothalamus, while potentially also influencing ovarian steroidogenesis (72). The DE mRNAs in the hypothalamus exhibited unique enrichment in steroid biosynthesis and Parathyroid hormone synthesis, secretion, and action pathways, emphasizing its dual function in neurotransmitter production and calcium homeostasis regulation. The pituitary-ovary axis demonstrated functional synergy through two key pathways: Neuroactive ligand-receptor interaction, which directly mediates hormone signaling in both tissues (73), and ECM-receptor interaction, which indirectly supports tissue remodeling during folliculogenesis by regulating cell-matrix interactions (74). The DE mRNAs in the pituitary were specifically enriched in Cortisol synthesis and secretion, which emerged as a critical regulator of the HPG axis, fine-tuning reproductive hormone release through stress feedback mechanisms (75). In the ovary, DE mRNAs were exclusively enriched in the Hippo signaling pathway and signaling pathways regulating pluripotency of stem cells, underscoring their central role in gametogenesis and follicular reserve maintenance, with Hippo-mediated follicular atresia ensuring oocyte quality control (76, 77).

To further clarify the interaction relationships among the DE RNAs, we constructed pairwise interaction networks for lncRNAs-mRNAs and for miRNAs-mRNAs, along with a ceRNA regulatory network involving all three types of RNA. In the hypothalamus, we identified the *GATA4* gene as a significant node appearing in both the lncRNA-mRNA and miRNA-mRNA interaction networks, as well as in the ceRNA regulatory network. The *GATA4* gene, encoding a key hypothalamic transcription factor, regulates basal GnRH expression by binding to its enhancer region and coordinating transcription in synergy with OCT1/CEBPB (78). In mice, the conditional knockout of *GATA4* in hypothalamic neurons and glia leads to reproductive abnormalities, mimicking conditions such as polycystic ovary syndrome (PCOS) (79). Additionally, we found that the lncRNAs-*miR-448-y-PMCH* ceRNA axis may also be a key regulator of hypothalamic function. The *PMCH* gene encodes the precursor for melanin-concentrating hormone (MCH), a hypothalamus-derived neuropeptide that functions as a key regulator of energy homeostasis, sleep architecture, and reward-related behaviors (80, 81). Studies have revealed that E_2_ reduces hypothalamic MCH neuron counts and serum MCH levels, while progesterone receptor blockade elevates MCH expression (82), indicating that reproductive hormones tightly regulate MCH expression to coordinate energy balance with reproductive demands.

In the pituitary gland, analysis of the lncRNA-mRNA interaction network revealed that *SMAD3* and *LHX3* are directly associated with pituitary function, that of the miRNA-mRNA interaction network identified *FOXL2* as functionally relevant, and that of the ceRNA regulatory network implicated *POU1F1* and *INHBA* in pituitary regulation. *SMAD3* mediates activin-induced transcription of the gene encoding the FSHβ subunit, which is essential for gonadal development and function (83). Additionally, *SMAD3* regulates follistatin gene transcription in cooperation with *FOXL2*, further influencing activin bioavailability and FSH synthesis (84). *INHBA* encodes the inhibin βA subunit, which forms activin A, a key regulator of FSH synthesis in pituitary gonadotropes. Activin A stimulates *Fshb (follicle stimulating hormone beta)* transcription through *SMAD3* and *FOXL2* (85), while also regulating follistatin expression, thereby creating a feedback loop that modulates activin bioavailability (86). PRL, regulated by *POU1F1*, is essential for lactation and has inhibitory effects on GnRH secretion, which can impact reproductive cycles (87). In mice, *LHX3* mutations lead to significant decreases in LH and FSH expression, resulting in hypogonadotropic hypogonadism (88). Within the ovarian lncRNA-mRNA interaction network, we identified *SPP1* and *COLQ* as genes associated with ovarian function. Concurrently, *SNAP25* and *AMPH* emerged as key components in the miRNA-mRNA regulatory axis. Notably, *SNAP25* was also detected as a target gene in the ceRNA network, suggesting that it has a multifaceted role in ovarian regulatory processes. In PCOS, *SPP1* is highly expressed in granulosa cells and is associated with monocyte activation, which can lead to ovarian fibrosis (89). Additionally, *SPP1*, a target of SOX3, acts as a key signaling node that activates the PI3K/AKT pathway, thereby promoting granulosa cell proliferation and inhibiting apoptosis. This suggests that *SPP1* may exert a promotive effect on female reproductive capacity (90). In PCOS, reduced expression of *SNAP25* is associated with impaired exocytosis in granulosa cells, which may contribute to the pathophysiology of the disease (91). *COLQ* is primarily associated with the assembly of acetylcholinesterase tetramers, and its involvement in protein assembly and cellular processes suggests that it may potentially indirectly affect ovarian health (92). *AMPH* is expressed in endocrine cells and is involved in synaptic vesicle dynamics. While *AMPH* was not shown to directly affect ovarian function, its expression in endocrine tissues suggests that it may be involved in hormonal regulation, which could indirectly impact ovarian function (93).

All the genes discussed above, along with their interacting DE lncRNAs and DE miRNAs, play essential roles in the HPO axis. Notably, additional genes that were noted to be highly expressed or highly connected in this axis, including *FAM189A2*, *FAM135B*, *YBX3*, and *TNFRSF13B*, along with their regulatory networks, demonstrate potential for modulating HPO axis activity.

## 5 Conclusion

This study systematically elucidated the molecular regulatory landscape of the HPO axis in Bian chickens, identifying tissue-specific differential expression patterns for lncRNAs, miRNAs, and mRNAs between the low-yield and high-yield groups. GO function and KEGG pathway analyses revealed key biological processes (cell proliferation, metabolic regulation, transport) and signaling cascades (progesterone-mediated oocyte maturation, steroid biosynthesis, Hippo signaling) underlying reproductive performance in the chicken. The integration of lncRNA, miRNA, and mRNA networks highlighted critical regulatory interactions targeting reproduction-related genes (e.g., *GATA4, SMAD3, FOXL2*), providing novel molecular markers for improving poultry egg-laying efficiency via targeted breeding strategies. These findings advance our understanding of chicken reproductive regulation and offer actionable targets for breeding.

## Data Availability

The raw sequence data reported in this paper have been deposited in the Genome Sequence Archive (Genomics, Proteomics and Bioinformatics 2021) in National Genomics Data Center (Nucleic Acids Res 2022), China National Center for Bioinformation/Beijing Institute of Genomics, Chinese Academy of Sciences (GSA: CRA029275 and GSA: CRA029278) that are publicly accessible at https://ngdc.cncb.ac.cn/gsa.
